# Ergodic Capacity of NOMA-Based Multi-Antenna LMS Systems with Imperfect Limitations

**DOI:** 10.3390/s22010330

**Published:** 2022-01-02

**Authors:** Haifeng Shuai, Rui Liu, Shibing Zhu, Changqing Li, Yi Fang

**Affiliations:** 1 School of Space Information, Space Engineering University, Beijing 101416, China; shuaihf99@163.com (H.S.); sbz_zhu@sohu.com (S.Z.); lcqqcl5577@sohu.com (C.L.); 2 Northern Institute of Electronics, Beijing 100089, China; fangyi950129@163.com

**Keywords:** land mobile satellite (LMS) systems, non-orthogonal multiple access (NOMA), imperfect limitations, ergodic capacity

## Abstract

With the rapid development of land mobile satellite (LMS) systems, large scale sensors and devices are willing to request wireless services, which is a challenge to the quality of service requirement and spectrum resources utilization on onboard LMS systems. Under this situation, the non-orthogonal multiple access (NOMA) is regarded as a promising technology for improving spectrum efficiency of LMS systems. In this paper, we analyze the ergodic capacity (EC) of NOMA-based multi-antenna LMS systems in the presence of imperfect limitations, i.e., channel estimation errors, imperfect successive interference cancellation, and co-channel interference. By considering multiple antennas at the satellite and terrestrial sensor users, the closed-form expression for EC of the NOMA-based LMS systems with imperfect limitations is obtained. Monte Carlo simulations are provided to verify theoretical results and reveal the influence of key parameters on system performance.

## 1. Introduction

In recent years, with the rapid development and significant demand of Internet of Things (IoT), telemedicine, Internet of Vehicles (IoV), and other technologies, land mobile satellite (LMS) systems have received tremendous attention from both academic and industry areas [1,2,3]. LMS systems can provide the high-throughput content transmission of a large number of terrestrial sensor users and devices with wide coverage [4,5,6]. Consequently, LMS systems have been the most effective method for re-establishing the links of communication after encountering natural disasters, such as earthquakes and typhoons [7,8].

Quality of service (QoS) and spectral efficiency are two vital constraints of the further development of LMS systems [9]. In this regard, a non-orthogonal multiple access (NOMA) scheme has been adopted in LMS systems to improve spectral efficiency and enhance the experience of served sensor users and devices [10,11]. NOMA can improve the spectral efficiency by transmitting content to multiple sensor users in the same time slot or frequency and improve the QoS of sensor users based on a power allocation factor by using power domain multiple access technology, which differs from the traditional orthogonal multiple access (OMA) scheme [12,13]. Due to the superiority of NOMA scheme, many studies have analyzed the performance improvement by applying NOMA technology to satellite communication (SatCom) systems. The authors of [14] investigated the outage performance of the NOMA-assisted satellite-terrestrial networks (STNs), where the closed-form analytical expression and asymptotic expression for the outage probability (OP) of the considered network were derived. An optimized power allocation model for NOMA-based SatCom systems is established to enhance QoS of sensor users in [15]. In [16], the authors applied NOMA scheme to integrated satellite-terrestrial content delivery (IST-CD) networks, and the analytical expressions for OP and hit probability were derived.

Furthermore, compared with single-antenna assisted NOMA systems, when multi-antenna is applied into the systems, the system’s capacity will be enhanced [17,18]. In [19], the authors investigated the performance of a integrated satellite-terrestrial relay network (ISTRN) with multi-antenna in each node and proved the positive impacts of multi-antenna techniques. The authors of [20] analyzed OP and throughout performance of a multi-antenna integrated satellite-terrestrial cooperative network. In [21], the authors discussed the reliability and security of a multi-antenna integrated satellite-terrestrial network (ISTN) and validated the superiority of multiple antennas scenario by using numerical results.

It is worth mentioning that many previous studies have adopted ideal system models [16,18]. Nevertheless, considering the impact of the practical situations, the NOMA-based LMS system is commonly affected by various imperfect limitations in the process of transmission and detection [22,23]. During the transmission process, many researchers assumed that the ideal channel state information (CSI) can be obtained [19]. However, in actual conditions, the channels usually experience severe fading, such as rain, fog, and other climatic effects; hence, it is difficult to obtain perfect CSI [24]. In fact, channel estimation errors (CEEs) actually occurred during channel estimation processing [25]. The authors of [26] considered imperfect CSI in a LMS systems, where the exact and asymptotic outage behavior of the system were obtained. In [27], the authors established a SatCom system model under non-ideal CSI and obtained optimal energy utilization efficiency of the system. In the process of signal detection, the NOMA scheme uses successive interference cancellation (SIC) technology to obtain the target signal of each sensor user in the superimposed signal [28,29]. However, due to the limitations of receiver performance, perfect SIC is difficult to achieve in reality [30,31]. The imperfect SIC situation in the NOMA-based SatCom was analyzed in [32], and the analytical expressions for OP of each sensor user and the corresponding asymptotic OP were derived. However, the authors selected an independent interference factor to represent the effects of imperfect SIC, which is lack of theoretical analysis. In addition, one more limitation is co-channel interference (CCI) due to the reuse of spectrum resources [33]. In [34], the authors evaluated the impacts of CCI on the LMS systems from the perspective of ergodic capacity (EC), OP, average symbol error rate (ASER), and energy efficiency (EE), respectively.

Motivated by these obervations, to the best of our knowledge, the ergodic capacity of the NOMA-based LMS systems with imperfect limitations, i.e., CEEs, imperfect SIC, and CCI, has not been investigated.

In particular, considering the above-mentioned realistic limitations, the major contributions of our work can be summarized as follows:

- Firstly, we illustrate a prevailing model for NOMA-based LMS systems under imperfect limitations. Imperfect SIC is analyzed due to the constraints of the receivers capability. Moreover, CEEs are considered owing to the imperfect CSI of the considered system. Along with the CEEs, another imperfect system limitations is considered due to frequency sharing, namely, CCI.
- Secondly, the analytical expression for EC of considered NOMA-based LMS systems is derived to evaluate the impacts of imperfect limitations on the performance of considered system. Moreover, the effects of CEEs, imperfect SIC, and CCI are revealed by theoretical results.
- Finally, the simulation results are further obtained to verify the efficiency and correctness of the theoretical results for the considered networks.

The remainder of this paper is arranged as follows. Section 2 provides the system model and forms the problems. Section 3 presents the statistical properties of the channels for satellite-terrestrial links and terrestrial-terrestrial links, and the closed-form analytical expression of EC for the considered system is derived under the imperfect limitations. Section 4 plots MC simulations results and details the theoretical analysis. Finally, Section 5 concludes the entire study.

Notations: Bold uppercase letters indicate matrices and bold lowercase letters indicate vectors. $\left|a\right|$ represents the absolute value of a complex scalar *a*. $E\left(b\right)$ stands for the expectation operator of *b*. $J_{v}\left(\cdot \right)$ represents the first-kind bessel function of order *v* ([35], Eq. 8.402). ${}_{1}F_{1}\left(c;d;z\right)$ denotes the confluent hypergeometric function ([35], Eq. 9.210.1). $G_{p,q}^{m,n}\left(\cdot \left|\cdot \right.\right)$ indicates Meijer-G function ([35], Eq. 9.301).

## 2. System Model

**Figure 1:**
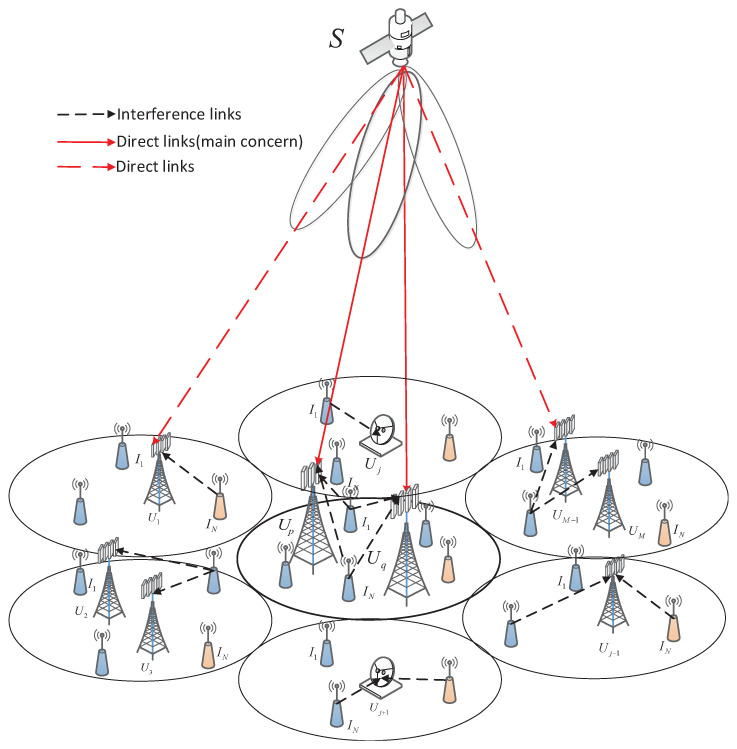
Illustration of a NOMA-based multi-antenna LMS system.

In this illustration shown as Figure 1, we consider a NOMA-based multi-antenna LMS system, where a geosynchronous earth orbit (GEO) satellite source (*S*) adopts the NOMA scheme to communicate with terrestrial sensor users $U_{j},j\in \left(1,\cdots ,N\right)$ through direct links. The main concern of this study is a two-user pair $U_{i},i\in \left(p,q\right)$ in one satellite beam. The two-user pair NOMA scenario has been recognized by the Third Generation Partnership Project (3GPP), which can enhance the spectrum efficiency of the sensor users [10]. Hence, we focus on the two-user pair NOMA scenario in order to simplify the model of the considered system and acquire a future perspective of the system’s performance, which is the basic research for the analysis of multiple sensor users NOMA situation [8]. Likewise, we assume that the two terrestrial sensor users are located in the same satellite beam of the satellite beams. Without loss of generality, the inter-beam interference is neglected by adopting four-color frequency reuse scheme [36], and the terrestrial sensor users $U_{i}$ are equipped with *M* antennas for improving antenna gain [18]. Moreover, by considering the co-use of the spectrum, multiple interference $I_{I},I\in \left\{1,2,\ldots ,N\right\}$ interfere with both sensor users [33].

### 2.1. Channel Model

In LMS systems, array fed reflector (AFR) technology reduces the processing consumption of on-boards by fixing the radiation mode of each antenna. Compared with direct radiating array (DRA) technology, the AFR technique can generate higher antenna gain and energy efficiency [15]. Considering the influence of free space loss (FSL), rain attenuation, and satellite antenna gain, the expression of the channel vector of satellite-terrestrial links is given by the following:

(1) $$\begin{array}{c} h_{SU_{i}}=\sqrt{F_{i}G_{i}}{\zeta_{i}}^{-\frac{1}{2}}\cdot f_{i}^{\frac{1}{2}}l_{SU_{i}}, \end{array}$$

with $F_{i}$ being the scale of FSL, which can be expressed as follows:

(2) $$\begin{array}{c} F_{i}={\left(\frac{V}{f}\right)}^{2}\frac{1}{d_{G}^{2}+d_{i}^{2}}, \end{array}$$

where V=cc4π4π, *c* represents the transmission speed of electromagnetic wave, *f* is the carrier frequency, $d_{G}\approx 35,786\mathrm{km}$ is the height of GEO satellite, and $d_{i}$ is the distance between terrestrial sensor user $U_{i}$ and the center of the satellite antenna coverage area center [6].

Then, $G_{i}$ represents the antenna gain of terrestrial sensor users $U_{i}$, which is given by the following:

(3) $$\begin{array}{c} G_{i}≃\left\{\begin{array}{cc} G_{\max}-2.5\times 10^{-3}{\left(\frac{d_{a}\theta }{\lambda }\right)}^{2}, & 0^{\circ }<\theta <\theta_{a}\\ 2+15\log\frac{d_{a}}{\lambda }, & \theta_{a}<\theta <\theta_{b}\\ 32-25\log\theta , & \theta_{b}<\theta <48^{\circ }\\ -10, & 48^{\circ }<\theta <180^{\circ } \end{array}\right., \end{array}$$

where $G_{\max}$ is the maximal gain of terrestrial sensor users antenna, $d_{a}$ is the diameter of the antenna, λ represents the wavelength of the signal, θ is the angle of off-boresight, and $\theta_{a}=\frac{20\lambda }{d_{a}}\sqrt{G_{\max}-\left(2+15\log\frac{d_{a}}{\lambda }\right)}$ and $\theta_{b}=15.85{\left(\frac{d_{a}}{\lambda }\right)}^{-0.6}$ represent the angle value, respectively.

Moreover, $\zeta_{i}={\left[\zeta_{i.1},\zeta_{i.2},\cdots ,\zeta_{i.M}\right]}^{T}$ represents the vector of rain attenuation. $\zeta_{i.m}$ follows lognormal random distribution, $\zeta_{i.m}∼CN\left(\mu ,\delta_{\zeta }^{2}\right)$, 1≤m≤M. In addition, $f_{i}={\left[f_{i.1},f_{i.2},\cdots ,f_{i.M}\right]}^{T}$ denotes the vector of the satellite antenna gain, and the expression of $f_{i.m}$ is given by the following:

(4) $$\begin{array}{c} f_{i,m}≃f_{\max}{\left(\frac{J_{1}\left(r_{i,m}\right)}{2r_{i,m}}+36\frac{J_{3}\left(r_{i,m}\right)}{r_{i,m}^{3}}\right)}^{2}, \end{array}$$

where $f_{\max}$ represents the maximal gain of satellite antenna, and $J_{1}\left(\cdot \right)$ and $J_{3}\left(\cdot \right)$ denote the first-kind bessel function of order 1 and 3, respectively. ri,m=τsinφi,mτsinφi,msinφ3dBsinφ3dB, τ=2.07123 denotes the angle coefficient, and $\varphi_{i,m}$ is the intersection angle between the beam center of the m-th antenna and the terrestrial sensor user $U_{i}$ relative to the satellite [18].

Finally, $l_{SU_{i}}$ denotes the random channel vector of satellite-terrestrial links. There are many mathematical models established for the satellite-terrestrial channel, such as Lutz, Markov, and Karasawa. In our paper, we adopt shadowed-Rician (SR) fading to describe the satellite-terrestrial channel [37]. The model of SR distribution is in good agreement with the actual measured data, and calculation complexity can be reduced at the same time. Thus, SR fading has been the popularly used channel model in LMS systems [19,24]. According to [37], the SR model of *m*-th component of $g_{SU_{i}}$ can be denoted as follows:

(5) $$\begin{array}{c} {\left(l_{SU_{i}}\right)}_{m}=Z\exp\left(j\varsigma \right)+A\exp\left(j\vartheta \right), \end{array}$$

where *Z* and *A* are all independent stationary random processes that represent the amplitudes of line-of-sight (LOS) and multi-path elements, and LOS and multi-path elements undergo Nakagami-m and Rayleigh distributions, correspondingly. ς is the deterministic phase of the LoS element. ϑ is the stationary random phase process, and it is uniformly distributed over [0,2π).

With the process of mathematical transformation, the probability density function (PDF) of the squared amplitude of $l_{SU_{i},m}$ is given by the following:

(6) $$\begin{array}{c} f_{{∥h_{SU_{i},m}∥}^{2}}\left(x\right)=\alpha_{SU_{i},m}{e^{-\beta_{SU_{i},m}x}}_{1}F_{1}\left(m_{SU_{i},m};1;\delta_{SU_{i},m}x\right), \end{array}$$

where αSUi,m=Δ2bSUi,mmSUi,m2bSUi,mmSUi,m+ΩSUi,mmSUi,m2bSUi,mmSUi,m2bSUi,mmSUi,m+ΩSUi,mmSUi,m2bSUi,m2bSUi,m, $\beta_{SU_{i},m}\overset{\Delta }{=}\frac{1}{2b_{SU_{i},m}}$, $\delta_{SU_{i},m}\overset{\Delta }{=}\frac{\Omega_{SU_{i},m}}{2b_{SU_{i},m}\left(2b_{SU_{i},m}m_{SU_{i},m}+\Omega_{SU_{i},m}\right)}$, $\Omega_{SU_{i},m},2b_{SU_{i},m}$, and $m_{SU_{i},m}$ represent the average power of the LOS element, the average power of the multi-path element, and the fading severity parameter with $m_{SU_{i},m}\in \left(0,\infty \right)$, respectively.

Assuming $m_{SU_{i},m}$ are integer values [9], after some algebraic manipulation [35], we can obtain the expression of the PDF as follows:

(7) $$\begin{array}{c} f_{{∥h_{SU_{i},m}∥}^{2}}\left(x\right)=\alpha_{SU_{i},m}\sum_{k=0}^{m_{SU_{i},m}-1}\zeta \left(k\right)x^{k}e^{-\left(\beta_{SU_{i},m}-\delta_{SU_{i},m}\right)x}, \end{array}$$

where $\zeta \left(k\right)={\left(-1\right)}^{k}{\left(1-m_{SU_{i},m}\right)}_{k}\delta_{SU_{i},m}^{k}/{\left(k!\right)}^{2}$ and ${\left(\cdot \right)}_{k}$ denotes the Pochhammer symbol [37].

### 2.2. Signal Model

*S* adopts the superposition coding technique (SCT) in order to send a superimposed signal to the two terrestrial sensor users $U_{i}$, which is given as follows:

(8) $$\begin{array}{c} x=\sqrt{a_{p}}s_{p}+\sqrt{a_{q}}s_{q}, \end{array}$$

where $s_{i}$ is the target signal of different $U_{i}$, satisfying $E\left[{\left|s_{i}\right|}^{2}\right]=1$. $a_{i}$ is the power allocation coefficient of different signals of the NOMA scheme, satisfying $a_{p}+a_{q}=1$. In this illustration, we design the channel of $U_{p}$, and it experiences more severe fading than $U_{q}$; hence, more power is allocated to the sensor users with worser channel conditions, i.e., $a_{p}>a_{q}$ [11]. As such, the signal received by $U_{i}$ is given by the following:

(9) $$\begin{array}{c} \begin{array}{c} y_{i}=h_{SU_{i}}w_{1}^{H}\sqrt{P_{S}}\left(\sqrt{a_{p}}s_{p}+\sqrt{a_{q}}s_{q}\right)+\sum_{I=1}^{N}g_{iI}w_{1}^{H}\sqrt{P_{I}}s_{iI}+w_{1}^{H}n_{i} \end{array}, \end{array}$$

where $P_{S}$ represents the transmitted power of satellite signal, $P_{S}=\sigma P$, *P* is the total power transmitted by the satellite, and σ is the power distribution coefficient with $\sigma \in \left(0,1\right)$. $h_{SU_{i}}$ denotes the channel vector between *S* and $U_{i}$ links, which undergoes SR fading [37]. $∥w_{1}∥=1$ is the beamforming (BF) vector. $s_{iI}$ is the interference signal from I. $P_{I}$ denotes the transmitting power of $I_{I}$. $g_{iI}$ represents the channel vector between the *I*-th interference and $U_{i}$ links, which are modeled as Rayleigh fading [38]. $n_{i}$ denotes addictive white Gaussian noise (AWGN) between the satellite and terrestrial sensor users modeled as $n_{i,m}∼CN\left(0,\delta_{i,m}^{2}\right)$. $\delta_{i,m}^{2}=K_{B}T_{i}$, $K_{B}=1.380649\times 10^{-23}J/K$ denotes the Boltzmann constant, and $T_{i}$ denotes the noise temperature of $U_{i}$.

### 2.3. Problem Formulation

Considering the practical system conditions, the electromagnetic environment and climate environment of the satellite-terrestrial links are extremely complex, resulting in the system being unable to obtain perfect CSI. Hence, the CEEs arise in the process of channel estimation [27]. Recalling the minimum mean square error (MMSE) method, the channel with CEEs is denoted as follows:

(10) $$\begin{array}{c} h_{SU_{i},m}={\tilde{h}}_{SU_{i},m}+e_{SU_{i},m}, \end{array}$$

where $h_{SU_{i},m}$ represents the actual amplitude of the SR fading channel of the satellite-terrestrial links, and ${\tilde{h}}_{SU_{i},m}$, and $e_{SU_{i},m}$ are the measured amplitude of SR fading channel and the channel estimation error of the satellite-terrestrial links, which are orthogonal to each other. In addition, the distribution of $e_{SU_{i}}$ is modeled as the complex Gaussian, $e_{SU_{i}}∼CN\left(0,{\bar{V}}_{e_{SU_{i},m}}\right)$. In this paper, we estimate the CSI of the satellite-terrestrial links by using estimation symbols [25]. Thus, the variance of $e_{SU_{i}}$ is expressed as follows:

(11) $$\begin{array}{c} \begin{array}{c} {\bar{V}}_{e_{SU_{i},m}}=E\left\{{\left|h_{SU_{i},m}\right|}^{2}\right\}-E\left\{{\left|{\tilde{h}}_{SU_{i},m}\right|}^{2}\right\}=\frac{1}{L_{SU_{i},m}{\bar{V}}_{SU_{i},m}+1} \end{array}, \end{array}$$

where $L_{SU_{i}}$ represents the quantity of the estimation symbols, ${\bar{V}}_{e_{SU_{i},m}}$ denotes the average signal-to-noise ratio (SNR) of the estimation symbols in the satellite-terrestrial links, and the expression of ${\bar{V}}_{SU_{i},m}$ can be written in the form of MMSE as follows:

(12) $$\begin{array}{c} {\bar{V}}_{SU_{i},m}=E\left\{V_{SU_{i},m}\right\}=P_{e}E\left\{{\left|h_{SU_{i},m}\right|}^{2}\right\}/\delta_{i,m}^{2}, \end{array}$$

where $P_{e}$ is the power of the estimation symbols, $P_{e}=\left(1-\sigma \right)P$. Currently, we use ${\bar{V}}_{e_{SU_{i},m}}$ to represent the accuracy of CEEs.

Moreover, maximal ratio combining (MRC) scheme is applied at the terrestrial sensor users to enhance the performance of the NOMA-based multi-antenna LMS system [21], which is expressed as follows.

(13) $$\begin{array}{c} w_{1}=\frac{h_{SU}}{{∥h_{SU}∥}^{2}}. \end{array}$$

Then, from (9) to (13), the signal-to-interference-plus-noise ratio (SINR) for detecting $s_{p}$ at $U_{q}$ is given by the following:

(14) $$\begin{array}{c} \gamma_{SU_{q-p}}=\frac{a_{p}\sigma \lambda_{SU_{q}}}{a_{q}\sigma \lambda_{SU_{q}}+\sigma {\bar{V}}_{e_{SU_{q}}}+\lambda_{l_{q}}+1}, \end{array}$$

where $\lambda_{SU_{q}}={\left|{\tilde{h}}_{SU_{q}}w_{1}^{H}\right|}^{2}P_{total}/\delta_{q}^{2}={\bar{\gamma }}_{SU_{q}}{∥{\tilde{h}}_{SU_{q}}∥}^{2}$, ${\bar{\gamma }}_{j},j\in \left\{SU_{p},SU_{q}\right\}$ denotes the average SNR of satellite to terrestrial sensor users links. $\lambda_{l_{q}}=\sum_{l=1}^{N}{\left|g_{lq}w_{1}^{H}\right|}^{2}P_{l}/\delta_{q}^{2}={\bar{\gamma }}_{q}\sum_{l=1}^{N}{∥g_{lq}∥}^{2}$, and ${\bar{\gamma }}_{i}$, $i\in \left\{p,q\right\}$ is the average SNR of different interference to $U_{i}$ links.

For the NOMA scheme, SIC technology is adopted to decode signal $x_{q}$ at terrestrial sensor user $U_{q}$ by eliminating the signal $x_{p}$. In this paper, imperfect SIC is considered at $U_{q}$ for practical limitations and the SINR by detecting $x_{q}$ is given by the following:

(15) $$\begin{array}{c} \gamma_{SU_{q}}=\frac{a_{q}\sigma \lambda_{SU_{q}}}{a_{p}\xi \sigma \lambda_{SU_{q}}+\sigma {\bar{V}}_{e_{SU_{q}}}+\lambda_{l_{q}}+1}, \end{array}$$

where ξ represents the relative coefficient of residual interference produced by imperfect SIC with $\xi \in \left(0,1\right)$.

Finally, we detect the signal $x_{p}$ at $U_{p}$ by assuming $x_{q}$ as an interference directly, and the SINR of signal $x_{p}$ is obtained as follows:

(16) $$\begin{array}{c} \gamma_{SU_{p}}=\frac{a_{p}\sigma \lambda_{SU_{p}}}{a_{q}\sigma \lambda_{SU_{p}}+\sigma {\bar{V}}_{e_{SU_{p}}}+\lambda_{l_{p}}+1}, \end{array}$$

where $\lambda_{SU_{p}}={\left|{\tilde{h}}_{SU_{p}}w_{1}^{H}\right|}^{2}P_{total}/\delta_{p}^{2}={\bar{\gamma }}_{SU_{p}}{∥{\tilde{h}}_{SU_{p}}∥}^{2}$ and $\lambda_{l_{p}}=\sum_{l=1}^{N}{\left|g_{lp}w_{1}^{H}\right|}^{2}P_{l}/\delta_{p}^{2}={\bar{\gamma }}_{p}\sum_{l=1}^{N}{∥g_{lp}∥}^{2}$.

It is worth noting that previous studies have only analyzed the special example of the imperfect system limitations. In our paper, firstly, we adopted estimation symbols to estimate CEEs, which is different from [25,27]. Moreover, CCI with Rayleigh fading is considered in our paper. Moreover, in [32], the interference caused by imperfect SIC is denoted as an independent factor. However, the stronger signal is taken into consideration under the imperfect SIC in our paper. Therefore, the above key elements in our manuscript are different from the more recently related works.

## 3. Performance Analysis

### 3.1. Preliminary Results

Before investigating the detailed performance of the considered system, we first obtain the statistical characteristics of the different links in the NOMA-assisted multi-antenna LMS systems under imperfect limitations.

The channel of the satellite-terrestrial links is modeled as SR fading [37]. According to (7) and considering MRC scheme, the PDF of $\gamma_{j}=\bar{\gamma_{j}}{\left|h_{j}\right|}^{2}$ is obtained as follows:

(17) $$\begin{array}{c} f_{\gamma_{j}}\left(x\right)=\frac{\alpha_{j}^{M}}{\Gamma \left(M\right)}\sum_{k=0}^{M\left(m_{j}-1\right)}\frac{\zeta {\left(k\right)}_{M}}{{\left({\bar{\gamma }}_{j}\right)}^{k+M}}x^{k+M-1}e^{-\Delta_{j}x}, \end{array}$$

where Δj=βj−δjβj−δjγ¯jγ¯j.

After some mathematical transformation steps [35], the cumulative distribution function (CDF) of $\gamma_{j}$ is given by the following.

(18) $$\begin{array}{c} \begin{array}{c} F_{\gamma_{j}}\left(x\right)=1-\frac{\alpha_{j}^{M}}{\Gamma \left(M\right)}\sum_{k_{j}=0}^{Mm_{j}-1}\sum_{t=0}^{k_{j}}\frac{{\left(M\left(1-m_{j}\right)\right)}_{k_{1}}{\left(-\delta_{j}\right)}^{k_{j}}}{k_{j}!{\left({\bar{\gamma }}_{j}\right)}^{k_{1}+M}t!\Delta_{j}^{k_{j}-t+M}}x^{t}e^{-\Delta_{j}x} \end{array}. \end{array}$$

The channel of the terrestrial links are assumed to undergo Rayleigh fading in this study [38]; hence, the PDF and CDF of SNR of CCI from the terrestrial transmitters to the terrestrial sensor users $\gamma_{i}$ are, respectively, expressed as follows:

(19) $$\begin{array}{c} f_{\gamma_{i}}\left(x\right)=\sum_{s=1}^{\varrho \left(A\right)}\sum_{n=1}^{\tau_{l}\left(A\right)}\chi_{s,n}\left(A\right)\frac{\mu_{\langle s\rangle }^{-n}}{\left(n-1\right)!}x^{n-1}e^{-\frac{x}{\mu_{\langle s\rangle }}}, \end{array}$$

(20) $$\begin{array}{c} F_{\gamma_{i}}\left(x\right)=1-\sum_{s=1}^{\varrho \left(A\right)}\sum_{n=1}^{\tau_{l}\left(A\right)}\sum_{k=0}^{n-1}\frac{\chi_{s,n}\left(A\right)}{k!}{\left(\frac{x}{\mu_{\langle s\rangle }}\right)}^{k}e^{-\frac{x}{\mu_{\langle s\rangle }}}, \end{array}$$

where $A=diag\left(\mu_{1},\mu_{2},\ldots ,\mu_{L}\right)$, $L\in \left\{N,M\right\}$, ${\left\{\mu_{s}\right\}}_{s=1}^{L}$ is the average SNR of the CCI links, $\varrho \left(A\right)$ is the quantity of distinct diagonal elements of A, $\mu_{\langle 1\rangle }>\mu_{\langle 2\rangle }>\ldots >\mu_{\langle \varrho \left(A\right)\rangle }$ are the distinct diagonal elements on decreasing sequence, $\tau_{l}\left(A\right)$ is the multiplicity of $\mu_{\langle s\rangle }$, and $\chi_{s,n}\left(A\right)$ is the $\left(s,n\right)th$ characteristic coefficient of A.

### 3.2. Ergodic Capacity Analysis

EC is a vital element that is often used to evaluate the performance of LMS systems. EC means the upper bound of the time average capacity on the all channels of the entire communication network [39]. In this illustration, we define the EC of the NOMA-based multi-antenna LMS system as the the sum of average instantaneous mutual information of the SINR of different terrestrial receivers [40], which is expressed as follows.

(21) $$\begin{array}{c} \begin{array}{c} EC_{SU}=\left\{E\left[\log_{2}\left(1+\gamma_{SU_{q-p}}\right)\right]+E\left[\log_{2}\left(1+\gamma_{SU_{q}}\right)\right]\right.\left.+E\left[\log_{2}\left(1+\gamma_{SU_{p}}\right)\right]\right\} \end{array}. \end{array}$$

By substituting (14)–(16) into (21), after some mathematical transformation steps, we can obtain the expression of EC at the below of this page.

(22) $$\begin{array}{c} \begin{array}{c} \begin{array}{c} EC_{SU}=\frac{1}{ln2}\;\\ \times \left\{E\left[\ln\left(\frac{\sigma \lambda_{SU_{2}}}{\sigma {\bar{\gamma }}_{SU_{2}}{\bar{V}}_{e_{SU_{2}}}+1}+\frac{\lambda_{l_{2}}}{\sigma {\bar{\gamma }}_{SU_{2}}{\bar{V}}_{e_{SU_{2}}}+1}+1\right)\right]-E\left[\ln\left(\frac{a_{2}\sigma \lambda_{SU_{2}}}{\sigma {\bar{\gamma }}_{SU_{2}}{\bar{V}}_{e_{SU_{2}}}+1}+\frac{\lambda_{l_{2}}}{\sigma {\bar{\gamma }}_{SU_{2}}{\bar{V}}_{e_{SU_{2}}}+1}+1\right)\right]\right. \end{array}\;\\ +E\left[\ln\left(\frac{\left(a_{2}+\xi a_{1}\right)\sigma \lambda_{SU_{2}}}{\sigma {\bar{\gamma }}_{SU_{2}}{\bar{V}}_{e_{SU_{2}}}+1}+\frac{\lambda_{l_{2}}}{\sigma {\bar{\gamma }}_{SU_{2}}{\bar{V}}_{e_{SU_{2}}}+1}+1\right)\right]-E\left[\ln\left(\frac{\xi a_{1}\sigma \lambda_{SU_{2}}}{\sigma {\bar{\gamma }}_{SU_{2}}{\bar{V}}_{e_{SU_{2}}}+1}+\frac{\lambda_{l_{2}}}{\sigma {\bar{\gamma }}_{SU_{2}}{\bar{V}}_{e_{SU_{2}}}+1}+1\right)\right]\;\\ \left.+E\left[\ln\left(\frac{\sigma \lambda_{SU_{1}}}{\sigma {\bar{\gamma }}_{SU_{1}}{\bar{V}}_{e_{SU_{1}}}+1}+\frac{\lambda_{l_{1}}}{\sigma {\bar{\gamma }}_{SU_{1}}{\bar{V}}_{e_{SU_{1}}}+1}+1\right)\right]-E\left[\ln\left(\frac{a_{2}\sigma \lambda_{SU_{1}}}{\sigma {\bar{\gamma }}_{SU_{1}}V_{e_{SU_{1}}}+1}+\frac{\lambda_{l_{1}}}{\sigma {\bar{\gamma }}_{SU_{1}}{\bar{V}}_{e_{SU_{1}}}+1}+1\right)\right]\right\} \end{array}. \end{array}$$

In order to simplify analysis, the following variable substitutions are applied: $A_{1}=\frac{\sigma }{\sigma {\bar{V}}_{e_{SU_{2}}}+1}$, $A_{2}=\frac{a_{2}\sigma }{\sigma {\bar{V}}_{e_{SU_{2}}}+1}$, $A_{3}=\frac{\left(a_{2}+\xi a_{1}\right)\sigma }{\sigma {\bar{V}}_{e_{SU_{2}}}+1}$, $A_{4}=\frac{\xi a_{1}\sigma }{\sigma {\bar{V}}_{e_{SU_{2}}}+1}$, $A_{5}=\frac{\sigma }{\sigma {\bar{V}}_{e_{SU_{1}}}+1}$, $\frac{a_{2}\sigma }{\sigma {\bar{V}}_{e_{SU_{1}}}+1}=A_{6}$, $B_{1}=\frac{1}{\sigma {\bar{V}}_{e_{SU_{2}}}+1}$, and $B_{2}=\frac{1}{\sigma {\bar{V}}_{e_{SU_{1}}}+1}$. The expression of $EC_{SU}$ can be written as (23) at the bottom of this page.

(23) $$\begin{array}{c} \begin{array}{c} \begin{array}{c} EC_{SU}=\frac{1}{ln2}\left\{E\left[\ln\left(A_{1}\lambda_{SU_{2}}+B_{1}\lambda_{l_{2}}+1\right)\right]-E\left[\ln\left(A_{2}\lambda_{SU_{2}}+B_{1}\lambda_{l_{2}}+1\right)\right]\right.\;\\ \;\;\;\;\;\;\;\;\;\;\;\;\;\;\;\;\;\;\;\;\;\;\;\;\;+E\left[\ln\left(A_{3}\lambda_{SU_{2}}+B_{1}\lambda_{l_{2}}+1\right)\right]-E\left[\ln\left(A_{4}\lambda_{SU_{2}}+B_{1}\lambda_{l_{2}}+1\right)\right] \end{array}\;\\ \;\;\;\;\;\;\;\;\;\;\;\;\;\;\;\;\;\;\;\;\;\;\;\;\;\;\;\;\;\;\;\left.+E\left[\ln\left(A_{5}\lambda_{SU_{1}}+B_{2}\lambda_{l_{1}}+1\right)\right]-E\left[\ln\left(A_{6}\lambda_{SU_{1}}+B_{2}\lambda_{l_{1}}+1\right)\right]\right\} \end{array}. \end{array}$$

Furthermore, let $\lambda_{SU_{q}}=x_{1}$, $\lambda_{l_{2}}=y_{1}$, $A_{1}\lambda_{SU_{q}}+B_{1}\lambda_{l_{2}}=z_{1}$. After some calculation, we can obtain the following.

(24) $$\begin{array}{c} f_{z_{1}}\left(z\right)=\int_{0}^{\infty }\frac{1}{A_{1}B_{1}}f_{Y}\left(\frac{z-u}{B_{1}}\right)f_{X}\left(\frac{u}{A_{1}}\right)du. \end{array}$$

By utilizing the expressions of SR fading (19) and Rayleigh fading (17) into (24), we can obtain the following.

(25) $$\begin{array}{c} \begin{array}{c} f_{z_{1}}\left(z\right)=\frac{\alpha_{{}_{SU_{q}}}^{M}}{M}\sum_{k=0}^{M\left(m_{SU_{q}}-1\right)}\frac{{\left(M\left(1-m_{SU_{q}}\right)\right)}_{k}{\left(-\delta_{SU_{q}}\right)}^{k}}{{\left(k!\right)}^{2}{\left({\bar{\gamma }}_{SU_{q}}\right)}^{k+1}}\;\\ \times \sum_{s=1}^{\varrho \left(A\right)}\sum_{n=1}^{\tau_{l}\left(A\right)}\sum_{t=0}^{n-1}\frac{{\left(-1\right)}^{t}\chi_{s,n}\left(A\right)\mu_{\langle s\rangle }^{-n}}{\left(n-1\right)!}\left(\begin{array}{c} n-1\\ t \end{array}\right)\left(k+t\right)!\;\\ \times \frac{1}{A_{1}^{k+1}B_{1}^{n}}{\left(\frac{\Delta_{SU_{q}}}{A_{1}}-\frac{1}{B_{1}\mu_{\langle s\rangle }}\right)}^{-\left(k+t\right)-1}z^{n-1-t}e^{-\frac{z}{B_{1}\mu_{\langle s\rangle }}} \end{array}. \end{array}$$

In what follows, let $\lambda_{SU_{p}}=x_{2}$, $\lambda_{l_{1}}=y_{2}$, $A_{2}\lambda_{SU_{q}}+B_{1}\lambda_{l_{2}}=z_{2}$, $A_{3}\lambda_{SU_{q}}+B_{1}\lambda_{l_{2}}=z_{3}$, $A_{4}\lambda_{SU_{q}}+B_{1}\lambda_{l_{2}}=z_{4}$, $A_{5}\lambda_{SU_{p}}+B_{2}\lambda_{l_{1}}=z_{5}$ and $A_{6}\lambda_{SU_{p}}+B_{2}\lambda_{l_{1}}=z_{6}$. We can obtain the related PDFs, respectively.

Moreover, the EC of the considered system can be rewritten as follows.

(26) $$\begin{array}{c} \begin{array}{c} EC_{SU}=\frac{1}{\ln2}\left\{\underset{E_{1}}{\underset{︸}{\int_{0}^{\infty }\ln\left(z+1\right)f_{z_{1}}\left(z\right)dz}}-\underset{E_{2}}{\underset{︸}{\int_{0}^{\infty }\ln\left(z+1\right)f_{z_{2}}\left(z\right)dz}}+\underset{E_{3}}{\underset{︸}{\int_{0}^{\infty }\ln\left(z+1\right)f_{z_{3}}\left(z\right)dz}}\right.\\ -\underset{E_{4}}{\underset{︸}{\int_{0}^{\infty }\ln\left(z+1\right)f_{z_{4}}\left(z\right)dz}}\left.+\underset{E_{5}}{\underset{︸}{\int_{0}^{\infty }\ln\left(z+1\right)f_{z_{5}}\left(z\right)dz}}-\underset{E_{6}}{\underset{︸}{\int_{0}^{\infty }\ln\left(z+1\right)f_{z_{6}}\left(z\right)dz}}\right\} \end{array}. \end{array}$$

With the help of ([41], Eq. 8.4.6.5), the Meijer-G function ([35], Eq. 9.301) is introduced to analysis EC as follows.

(27) $$\begin{array}{c} \ln\left(1+z\right)=G_{22}^{12}\left(z\left|\begin{array}{cc} 1, & 1\\ 1 & 0 \end{array}\right.\right). \end{array}$$

By substituting (27) and (25) into $E_{1}$, with the help of ([41], Eq. 2.24.3.1) and ([41], Eq. 8.2.2.14), $E_{1}$ can be expressed as (28).

(28) $$\begin{array}{c} \begin{array}{c} E_{1}=\frac{\alpha_{{}_{SU_{q}}}^{M}}{M}\sum_{k=0}^{M\left(m_{SU_{q}}-1\right)}\frac{{\left(M\left(1-m_{SU_{q}}\right)\right)}_{k}{\left(-\delta_{SU_{q}}\right)}^{k}}{{\left(k!\right)}^{2}{\left({\bar{\gamma }}_{SU_{q}}\right)}^{k+1}}\sum_{s=1}^{\varrho \left(A\right)}\sum_{n=1}^{\tau_{l}\left(A\right)}\frac{\chi_{s,n}\left(A\right)\mu_{\langle s\rangle }^{-t}}{\left(n-1\right)!}\sum_{t=0}^{n-1}\left(\begin{array}{c} n-1\\ t \end{array}\right){\left(-1\right)}^{t}\;\\ \times \left(k+t\right)!\frac{1}{A_{1}^{k+1}B_{1}^{t}}{\left(\frac{\Delta_{SU_{2}}}{A_{1}}-\frac{1}{B_{1}\mu_{\langle s\rangle }}\right)}^{-\left(k+t\right)-1}G_{32}^{13}\left(B_{1}\mu_{\langle s\rangle }\left|\begin{array}{c} 1-n+t,1,1\\ 1,0 \end{array}\right.\right) \end{array}. \end{array}$$

Applying the similar method, we can also obtain the expressions of $E_{2}$, $E_{3}$, $E_{4}$, $E_{5}$, and $E_{6}$, correspondingly.

At last, by substituting the expressions of $E_{1}-E_{6}$ into (26), after a simple arrangement, the final expression of $EC_{SU}$ can be expressed as (29) at the top of this page.

(29) $$\begin{array}{c} \begin{array}{c} EC_{SU}=\frac{1}{\ln2}\frac{\alpha_{SU_{q}}^{M}}{M}\;\\ \times \sum_{k=0}^{M\left(m_{SU_{q}}-1\right)}\frac{{\left(M\left(1-m_{SU_{q}}\right)\right)}_{k}{\left(-\delta_{SU_{2}}\right)}^{k}}{{\left(k!\right)}^{2}{\left({\bar{\gamma }}_{SU_{2}}\right)}^{k+1}}\sum_{s=1}^{\varrho \left(A\right)}\sum_{n=1}^{\tau_{l}\left(A\right)}\sum_{t=0}^{n-1}\frac{{\left(-1\right)}^{t}\chi_{s,n}\left(A\right)}{\left(n-1\right)!\mu_{\langle s\rangle }^{t}}G_{32}^{13}\left(B_{1}\mu_{\langle s\rangle }\left|\begin{array}{c} 1-n+t,1,1\\ 1,0 \end{array}\right.\right)\;\\ \times \left(\begin{array}{c} n-1\\ t \end{array}\right)\left(k+t\right)!\left\{\frac{1}{A_{1}^{k+1}B_{1}^{t}}{\left(\frac{\Delta_{SU_{2}}}{A_{1}}-\frac{1}{B_{1}\mu_{\langle s\rangle }}\right)}^{-\left(k+t\right)-1}-\frac{1}{A_{2}^{k+1}B_{1}^{t}}{\left(\frac{\Delta_{SU_{2}}}{A_{2}}-\frac{1}{B_{1}\mu_{\langle s\rangle }}\right)}^{-\left(k+t\right)-1}\right.\;\\ \left.+\frac{1}{A_{3}^{k+1}B_{1}^{t}}{\left(\frac{\Delta_{SU_{2}}}{A_{3}}-\frac{1}{B_{1}\mu_{\langle s\rangle }}\right)}^{-\left(k+t\right)-1}-\frac{1}{A_{4}^{k+1}B_{1}^{t}}{\left(\frac{\Delta_{SU_{2}}}{A_{4}}-\frac{1}{B_{1}\mu_{\langle s\rangle }}\right)}^{-\left(k+t\right)-1}\right\}+\frac{1}{\ln2}\frac{\alpha_{SU_{p}}^{M}}{M}\;\\ \times \sum_{k=0}^{M\left(m_{SU_{p}}-1\right)}\frac{{\left(M\left(1-m_{SU_{p}}\right)\right)}_{k}{\left(-\delta_{SU_{1}}\right)}^{k}}{{\left(k!\right)}^{2}{\left({\bar{\gamma }}_{SU_{1}}\right)}^{k+1}}\sum_{s=1}^{\varrho \left(A\right)}\sum_{n=1}^{\tau_{l}\left(A\right)}\sum_{t=0}^{n-1}\frac{{\left(-1\right)}^{t}\chi_{s,n}\left(A\right)}{\left(n-1\right)!\mu_{\langle s\rangle }^{t}}G_{32}^{13}\left(B_{2}\mu_{\langle s\rangle }\left|\begin{array}{c} 1-n+t,1,1\\ 1,0 \end{array}\right.\right)\;\\ \times \left(\begin{array}{c} n-1\\ t \end{array}\right)\left(k+t\right)!\left\{\frac{1}{A_{5}^{k+1}B_{2}^{t}}{\left(\frac{\Delta_{SU_{2}}}{A_{5}}-\frac{1}{B_{2}\mu_{\langle s\rangle }}\right)}^{-\left(k+t\right)-1}-\frac{1}{A_{6}^{k+1}B_{2}^{t}}{\left(\frac{\Delta_{SU_{2}}}{A_{6}}-\frac{1}{B_{2}\mu_{\langle s\rangle }}\right)}^{-\left(k+t\right)-1}\right\} \end{array}. \end{array}$$

## 4. Numerical Results

In this section, we provide MC simulations to validate the performance of NOMA-based multi-antenna LMS systems under different situations. The simulation parameters of the considered system are provided in Table 1 [18]. SR fading channels parameters are shown in Table 2 [10]. Moreover, without loss of generality, we assume $\delta_{p}^{2}=\delta_{q}^{2}=\delta^{2}$, ${\bar{\gamma }}_{SU_{p}}={\bar{\gamma }}_{SU_{q}}=\bar{\gamma }$, ${\bar{\gamma }}_{1}={\bar{\gamma }}_{2}={\bar{\gamma }}_{I}$, $k_{SU_{p}}=k_{SU_{q}}=k$, and ${{}_{L}}_{SU_{p}}={{}_{L}}_{SU_{q}}={}_{L}$ [19,20].

**Figure 2:**
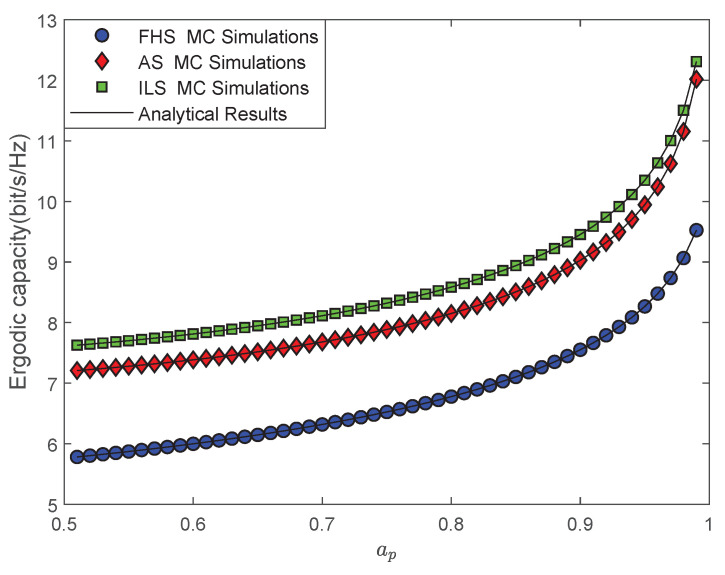
The EC versus $a_{p}$ for different SR fading channels.

Figure 2 illustrates the impacts of different SR fading channels on the EC of the NOMA-based multi-antenna LMS system under imperfect system limitations, where ξ=0.01,L=5,σ=0.75,N=1 and $\bar{\gamma_{2}}=1\;dB$. First of all, we can clearly observe that MC simulations are in excellent agreement with analytical results, which verifies the effectiveness of our theoretical analysis. Then, it can be clearly observed the EC of our considered system gradually becomes larger with the increase in $a_{p}$. However, from a practical point of view, the QoS and fairness of the terrestrial sensor user $U_{q}$ are degraded when $a_{p}$ tends to 1. In addition, the EC of $U_{q}$ is severely deteriorated owing to less power distribution. Based on the consideration of user farness, the value range of $a_{p}$ is usually selected in (0.7, 0.8). In the following simulations, we employ $a_{p}=0.75$ as the power allocation coefficient of the NOMA-based system.

**Figure 3:**
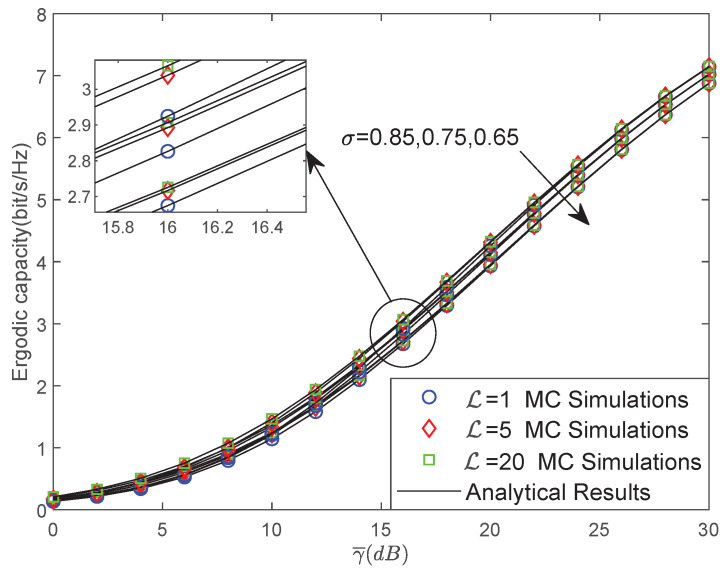
The EC versus $\bar{\gamma }$ for different CEEs situations.

Figure 3 examines the EC versus $\bar{\gamma }$ for different CEEs situations with L=1,5,20 and σ = 0.85, 0.75, 0.65. As the values of L and σ become larger, the CEEs of the NOMA-based LMS networks become smaller, which results in improved EC. Moreover, with the increase in the average SNR of the considered system, the impact of L becomes lighter. This observation can be explained when the system has high SNR, the system can obtain better CSI so as to reduce the impact of the errors during channel estimation processes.

**Figure 4:**
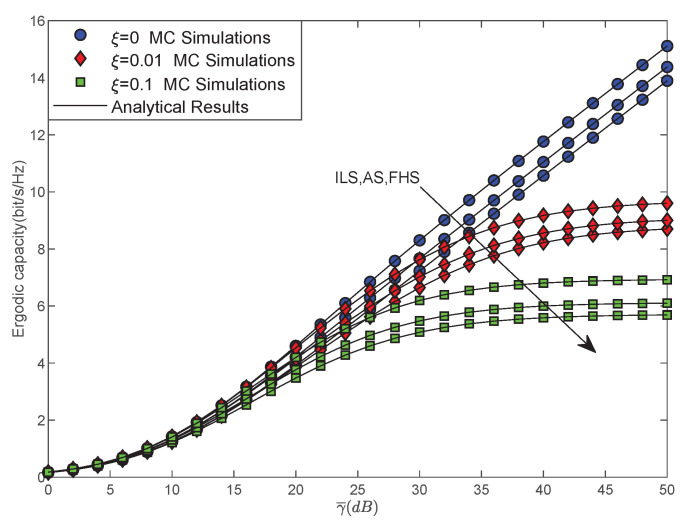
EC versus $\bar{\gamma }$ for different imperfect SIC situations.

Figure 4 plots the EC versus $\bar{\gamma }$ for different SR fading channels with different imperfect SIC coefficient ξ, where $\xi =\left\{0,0.01,0.1\right\}$, and ξ=0 represents the perfect SIC situation. From the figure, we can clearly find that the EC of the considered system enhances gradually along with the improvement of channel conditions. Moreover, we can find that EC is sensitive to the imperfect SIC coefficient. When ξ becomes larger, the EC of the considered network becomes worse. This is because NOMA sensor users apply SIC technology to detect the received signal at the receiving end. When the performance of the receivers is weak, SIC technology is imperfect, and the transmission performance of the system directly imposes a large negative impact, resulting in a reduction in EC for the system.

**Figure 5:**
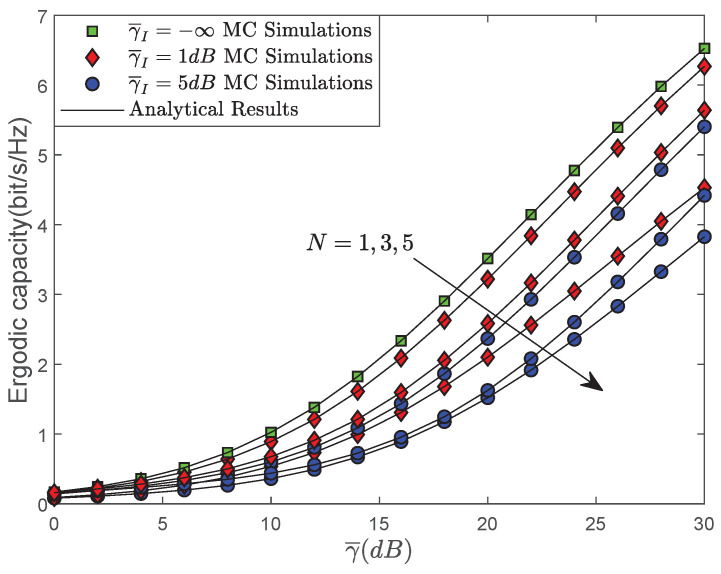
EC versus $\bar{\gamma }$ for different CCI situation.

Figure 5 depicts the EC versus $\bar{\gamma }$ for different CCI situations with N=1,3,5 and $\bar{\gamma_{I}}=\infty ,1,5$ dB. Among all scenarios, $\bar{\gamma_{I}}=\infty$ denotes that no CCI is considered in our system. As illustrated, we can first observe that the EC performance of the system has a noticeable degradation under the CCI scenario. Moreover, it can be found that the EC of the system degrades significantly with an increase in the power of interference and the quantity of interference, which is consistent with the realistic situation.

**Figure 6:**
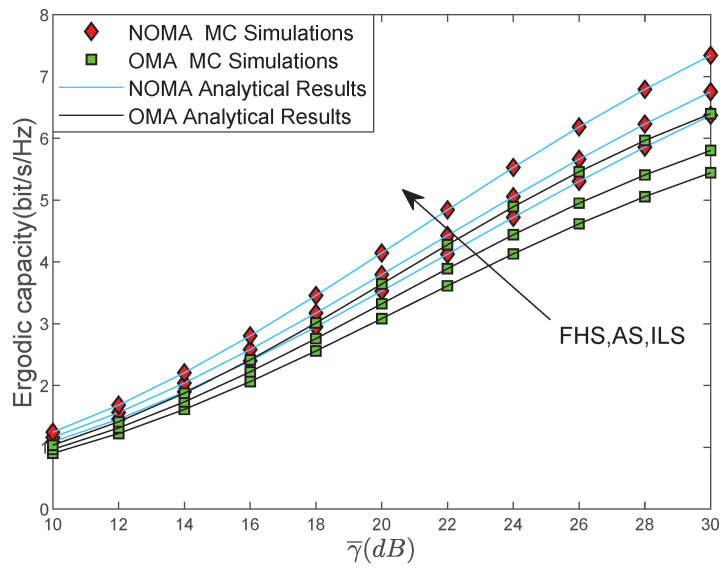
EC versus $\bar{\gamma }$ with NOMA and OMA.

Figure 6 compares EC versus $\bar{\gamma }$ with our proposed NOMA-based system and the traditional OMA-based system, i.e., Time Division multiple access (TDMA). As we can observe, the NOMA-based multi-antenna LMS system enhances EC performance when compared with the EC curves of benchmark traditional OMA-based system, which proves the superiority of the NOMA scheme.

## 5. Discussion

In this study, we have established a NOMA-based multi-antenna LMS system by considering practical conditions, including CEEs, imperfect SIC, and CCI. The imperfect limitations inevitably restricted EC performance of the NOMA-based multi-antenna LMS system. In order to reveal valuable insight into the influence of each imperfect limitations on the EC of the LMS system, we derived the analytical expression of the EC under imperfect limitations. Finally, MC simulations were implemented to validate the accurateness of our analytical derivations. The simulation results verified the impact of each imperfect limitations on the EC performance of the considered system and demonstrated the significance of practical networks with perfect system conditions.

## Figures and Tables

**Table 1:** System parameters.

Parameter	Value
Satellite Orbit	GEO
Carrier Frequency	18 GHz
Carrier Bandwidth	*B* = 50 MHz
3 dB angle	$\varphi_{3\mathrm{dB}}=0.4{}^{\circ}$
Maximal Beam Gain	$f_{max}$ = 48 dB
Receive Antenna Gain	${{}_{G}}_{max}$ = 4 dB
Noise Temperature	*T* = 300 K
Rain Attenuation	$\mu =-3.125,\delta_{\zeta }^{2}=1.591$

**Table 2:** Channel parameters.

Shadowing	$m_{j}$	$b_{j}$	$\Omega_{j}$
Frequent heavy shadowing (FHS)	1	0.063	0.0007
Average shadowing (AS)	5	0.251	0.279
Infrequent light shadowing (ILS)	10	0.158	1.29

## Abbreviations

The following abbreviations are used in this manuscript:

3GPP	Third Generation Partnership Project;
AFR	Array fed reflector;
AS	Average shadowing;
ASER	Average symbol error rate;
AWGN	Addictive white Gaussian noise;
CCI	Co-channel interference;
CDF	Cumulative distortion function;
CEEs	Channel estimation errors;
CSI	Channel state information;
DRA	Direct radiating array;
EC	Ergodic capacity;
EE	Energy efficiency;
FHS	Frequent heavy shadowing;
GEO	Geosynchronous earth orbit;
ILS	Infrequent light shadowing;
IoV	Internet of vehicles;
IoT	Internet of things;
IST-CD	Integrated satellite-terrestrial content delivery;
ISTN	Integrated satellite-terrestrial network;
ISTRN	Integrated satellite-terrestrial relay network;
LMS	Land mobile satellite;
LOS	Line-of-sight;
MC	Monte Carlo;
MMSE	Minimum mean square error;
MRC	Maximal Ratio Combining;
NOMA	Non-orthogonal multiple access;
OMA	Orthogonal multiple access;
OP	Outage probability;
PDF	Probability distribution function;
QoS	Quality of service;
SatCom	Satellite communication;
SCT	Superposition coding technique;
SIC	Successive interference cancellation;
SINR	Signal to interference plus noise ratio;
SNR	Signal-to-noise ratio;
SR	Shadowed-Rician;
STNs	Satellite-terrestrial networks.
